# Analysis of the Fecal Metabolomic Profile in Breast vs. Different Formula Milk Feeding in Late Preterm Infants

**DOI:** 10.3390/metabo14010072

**Published:** 2024-01-22

**Authors:** Giuseppe De Bernardo, Gilda D’Urso, Simona Spadarella, Maurizio Giordano, Giuseppina Leone, Agostino Casapullo

**Affiliations:** 1 Division of Pediatrics Neonatology and NICU, Ospedale Buon Consiglio Fatebenefratelli, 80123 Naples, Italy; spadarella.simona@fbfna.it (S.S.); leone.giuseppina@fbfna.it (G.L.); 2 Department of Pharmacy, University of Salerno, Via Giovanni Paolo II, 132, 84084 Fisciano, Italy; gidurso@unisa.it; 3 Department of Clinical Medicine and Surgery, Federico II University, 80138 Naples, Italy; mauri.giordano@studenti.unina.it

**Keywords:** newborn, neonatal nutrition, breast milk, postbiotic enrichment, *Lactobacillus paracasei*, mass spectrometry-based metabolomics, multivariate data analysis

## Abstract

Human milk is the gold standard for infant nutrition, but when it is not available or insufficient to satisfy the needs of the infant, formula milk is proposed as an effective substitute. A prospective observational cohort study was conducted on late preterm infants fed with breast and two different formula milks. On this basis, they were divided into three groups: group FMPB (fed with formula + postbiotic), group FM (fed with standard formula), and group BM (breastfed). Stool samples for a metabolomic study were collected at T0 (5–7 days after birth), T1 (30 days of life), and T2 (90 days of life), giving rise to 74 samples analyzed via liquid chromatography coupled with high-resolution mass spectrometry. The T0, T1, and T2 LC-MS raw data were processed for Partial Least Square Discriminant Analysis (PLS-DA), followed by a statistical analysis. This preliminary study highlighted a good overlapping between the fecal metabolome of breast and substitute feeding systems, confirming the efficacy of the formula preparations as breast milk substitutes. Moreover, several similarities were also detected between the FMPB and BM metabolome, highlighting that the addition of a postbiotic to standard formula milk could be more effective and considered a better alternative to breast milk.

## 1. Introduction

Balanced nutrition from early childhood significantly influences growth and psychomotor development. In contrast to conventional foods, functional foods have demonstrated physiological benefits and can reduce the risk of chronic disease beyond basic nutritional functions, including the maintenance of gut health [1]. Exclusively breast milk is suggested from birth to 6 months of life as the normative standard for an infant’s nutrition as it guarantees everything necessary for growth, maturation, protection from infections, and promoting the development of a balanced intestinal microbiota [2]. Human milk provides several bioactive components, like natural probiotics (*Bifidobacterium* spp. and *Lactobacillus* spp.) and their metabolites, which colonize the intestine of the newborn and exert a crucial role in the development of the gut immune system’s attenuating inflammatory processes. Several prenatal and perinatal factors, including the type of delivery, the use of antibiotics, diet, and other environmental factors, can influence the microbial colonization of the newborn [3]. It is generally accepted that the intestinal microbiota of the healthy, full-term, vaginally delivered, breastfed infant constitutes the gold standard for a favorable microbial composition in the first few years of life [4,5,6].

However, in cases where breast milk is unavailable or insufficient to satisfy the infant’s nutritional needs, formula milk is offered and used as a substitute. Given the benefits, it is essential that formula milk is as close as possible to human milk, providing bioactive substances that target gut and immune system health. Current research focuses on the optimization of artificial formulas, with the aim of resembling human milk in composition and functionality, with some products on the market already including probiotics, prebiotics, symbiotics, and postbiotics [5]. However, it remains to be clarified which is the best formulation for the best development of immune and microbial systems of newborns [7]. The study of the metabolomic profile of newborns could reveal the presence of metabolites with an important anti-inflammatory and antioxidant action and that could have beneficial effects on the newborn’s development. Postbiotics are preparations composed of both microbial constituents and their metabolites, produced during fermentation. It has already been highlighted in the literature that the enrichment of formula milk with postbiotics seems to offer advantages for the nutrition of full-term newborns, in the absence of breast milk, as it promotes immune, metabolic, and microbial maturation, like human milk, thus making postbiotics very promising and interesting supplements in the nutrition of newborns and infants. Mass spectrometry-based metabolomics is one of the best approaches to analyze the metabolic profile of complex biological samples, such as blood, urine, and stool, both in qualitative and quantitative terms. The application of high-resolution HPLC combined with conventional and tandem mass spectrometry experiments on instruments with a high resolving power, such as Orbitraps or FT-ICR, improved the coverage of the metabolite content and the accuracy of the results. Once data have been acquired, a second step is needed for their analysis, based on statistical approaches. The most-used projection methods include Principal Component Analysis (PCA) and Partial Least Square Discriminant Analysis (PLS-DA) and its variants. Metabolomics based on mass spectrometry is extremely useful in understanding the metabolic pathways regulated by the host–gut microbiota interaction. Of particular interest, stool samples are easily accessible and provide a non-invasive window to study the effect of the diet–gut microbiota–host interaction through the analysis of the remaining unabsorbed metabolites [7]. The innovation of our study was to analyze the metabolomic profile of late preterm newborns. Few studies including the population of premature newborns, in particular late preterm infants, have been reported so far. The latter have a gestational age between 34 + 0 and 36 + 6 weeks. They are frequently overlooked, due to their size in comparison to premature infants. Nonetheless, their susceptibility persists owing to physiological and structural immaturity. The primary aim of our study was to evaluate the metabolomic profiles of late preterm infants fed by breast milk, formula milk, or formula milk enriched with a postbiotic (SMART D3 MATRIX), at 5–7 days, 1 month, and 3 months of life using both targeted and pseudo-targeted MS-based metabolomics approaches. The preliminary results could confirm the efficacy of formula milk as a good alternative to breastfeeding, also highlighting that the addition of a postbiotic preparation to the formula milk could represent an alternative neonatal nutrition of the breast milk.

## 2. Materials and Methods

### 2.1. Study Design

A prospective observational cohort study was conducted at the Department of Woman and Child, Buon Consiglio Fatebenefratelli Hospital, Naples, Italy, from January 2022 to September 2023. This study was recorded and publicly accessible at https://classic.clinicaltrials.gov/ct2/show/NCT06052592, accessed on 13 January 2024. Inclusion criteria were late preterm newborns (gestational age between 34 + 0 and 36 + 6 weeks), appropriate weight for gestational age, written informed consent. Exclusion criteria were weight percentile < 10th and/or >90th, heart disease, liver disease, gastrointestinal diseases with malabsorption, endocrinological diseases, perinatal infections, metabolic and genetic diseases, born to mothers with endocrinological and metabolic diseases, insufficient sample, failure to obtain sample, and withdrawal of informed consent. At the time of birth, all mothers were encouraged to feed their sons via breastfeeding or, when human milk was not sufficient or not available, formula milk or formula milk and postbiotic was administered. Milk supplementation with postbiotic was obtained with 0.5 mL of SMART D3 MATRIX supplied free of charge by Smartfarma S.r.l. (Vitamin D3 10 mcg 400 I.U., Immunofos (fermented FOS from *Lactobacillus paracasei* strain CNCM I-5220) 20 mg). Newborns were enrolled to receive a standard formula (group FM) or a standard formula implemented with a postbiotic (group FMPB) in comparison with breast milk (BM, reference group), and the metabolic profile at 5–7 days (T0), 1 month (T1), and 3 months (T2) of life was analyzed. A stool sample of 20 g was collected from each newborn on the fifth day of life (T0), one month (T1), and three months of life (T2). The stool samples collected were transferred into test tubes, supplied by the experimental center, of adequate capacity and stored at −20 °C until the time of the study and they were evaluated using a mass spectrometry-based metabolomics approach on stool samples.

### 2.2. Sample Preparation

Each stool sample was extracted by following the protocol described by Zeng et al. [8]. A mixture of acetonitrile and water (2:1, *v*/*v*) was added to each sample (1 g feces: 10 mL of solvent), followed by vortex-mixing for 3 min and centrifuging at 10,000 rpm for 5 min at four °C. After being transferred into a fresh 1.5 mL tube and centrifuged again for 5 min at 10,000 rpm, the supernatant was filtered with PVDF filter 0.2 µm. Before LC-MS analysis, an aliquot of 100 µL of each extract was diluted in 100 µL of water (LC-MS grade). 

### 2.3. LC-MS Analysis 

Experiments were achieved using a Thermo scientific liquid chromatography system constituted of a quaternary Accela 600 pump and an Accela autosampler connected to a linear Trap-Orbitrap hybrid mass spectrometer (LTQ-Orbitrap XL, Thermo Fisher Scientific, Bremen, Germany) with electrospray ionization (ESI). Luna omega polar C18 column (150 × 2.1 mm), particle size 3 µm (Phenomenex Aschaffenburg, Germany), was used as the stationary phase, while acetonitrile/water (95:5) with 0.1% formic acid (B) and water/acetonitrile (95:5) with 0.1% formic acid (A) were used as mobile phases. A linear gradient program at a flow rate of 0.2 mL/min was used, starting from 5% of B and ending at 95%B in 30 min. An amount of 3 µL of each sample was injected. Analyses were performed in negative ion mode. ESI source parameters were as follows: capillary voltage −48 V; tube lens voltage −176.47; capillary temperature 280 °C; sheath and auxiliary gas flow (N_2_), 15 and 5; sweep gas 0; spray voltage 5. MS spectra were acquired via full-range acquisition covering *m*/*z* 180–1300. Xcalibur software version 2.1 was used for instrument control, data acquisition, and data analysis. For T0 and T1, 27 samples were analyzed in duplicates (54 samples in total). For T2, 20 samples were analyzed in duplicates (40 samples in total). Data-dependent acquisition mode was performed for MS fragmentation of the first and the second most intense ion from the FULL MS scan event. For this experiment, a collision energy at 30%, a minimum signal threshold of 300, an isolation width of 2.0, and a resolving power of 30,000 were used [9]. 

### 2.4. Multivariate Data Analysis 

#### 2.4.1. Untargeted Approach

LC-MS raw data of the T0, T1, and T2 samples were processed separately using the software mzmine 2.53 (http://mzmine.github.io/download.html, accessed on 13 January 2024), obtaining three data matrices (one for each time) composed by N observations represented by the analyzed samples (54 samples for T0 and T1, 40 samples for T2) and Y variables represented by the *m*/*z* value for each peak (631 peaks for T0 samples, 819 peaks for T1 samples, and 620 peaks for T2); further analysis of the data matrices was processed by SIMCA P + software 17.0 (Umetrix AB, Umea Sweden) for Partial Least Square Discriminant Analysis (PLS-DA) [2]. Log transformation and the Pareto scale were used. The quality of the model was determined by the R2 and Q2 values.

For PLS-DA, matrix X has the dimensions N×S, where N represents our observations, each analyzed sample, and S corresponds to the variables *m*/*z* with the peak area. Y (N × 1) is the vector containing the class that each sample belongs to, coded into 1 (formula plus postbiotic-fed infants), 0 (breastfed infants), and −1 (formula-fed infants). 

#### 2.4.2. Pseudo-Targeted Approach

Data analysis was performed using Compound Discoverer software version 3.3 (Thermo Fisher Scientific), a commercial software for the identification of small molecule structures. Raw data in full ms and data-dependent scan were uploaded on the software and processed with workflow “untargeted metabolomics with statistics detect unknowns with ID using online database”. Finally, a tentative list of identified metabolites was obtained. In this way, a new data matrix with detected metabolites was generated using the peak rating values for the three times; again, three new matrices generated for each time were processed by SIMCA P + software 17.0 (Umetrix AB, Umea, Sweden) for Partial Least Square Discriminant Analysis (PLS-DA).

### 2.5. Statistical Analysis

The sample size was calculated using G*Power 3.1.9.2 for Windows [10]. The sample size was calculated using Cohen’s rules of thumb due to missing data, estimating that there was a small effect size in the concentration of metabolome among the 3 groups. Cohen, in 1969, defined the following conventional effect sizes in the case of insufficient data to calculate the sample size without overestimating the power: small f = 0.14; medium f = 0.25; large f = 0.40 [11]. Setting effect size = 0.25, α = 0.05, β = 0.20, number of groups = 3, number of measurements = 3, the correlation between repeated measures = 0.7, correction for non-sphericity = 1, the sample size was 24. Statistical analysis was carried out by a statistician who was aware of the study aim using SPSS version 25.0 for Windows (IBM, Armonk, NY, USA). The normal distribution of data was evaluated via the Shapiro–Wilk test. Continuous data were reported as the median and interquartile range or median ± standard deviation, if they had normal distribution or non-normal distribution. Parametric data were explored via one-way ANOVA test. Nominal data were studied by chi square test and reported as percentages. Non-parametric data were analyzed via the Kruskal–Wallis test with the h Bonferroni post hoc test. All data with *p* < 0.05 were considered statistically significant.

## 3. Results

**Figure 1:**
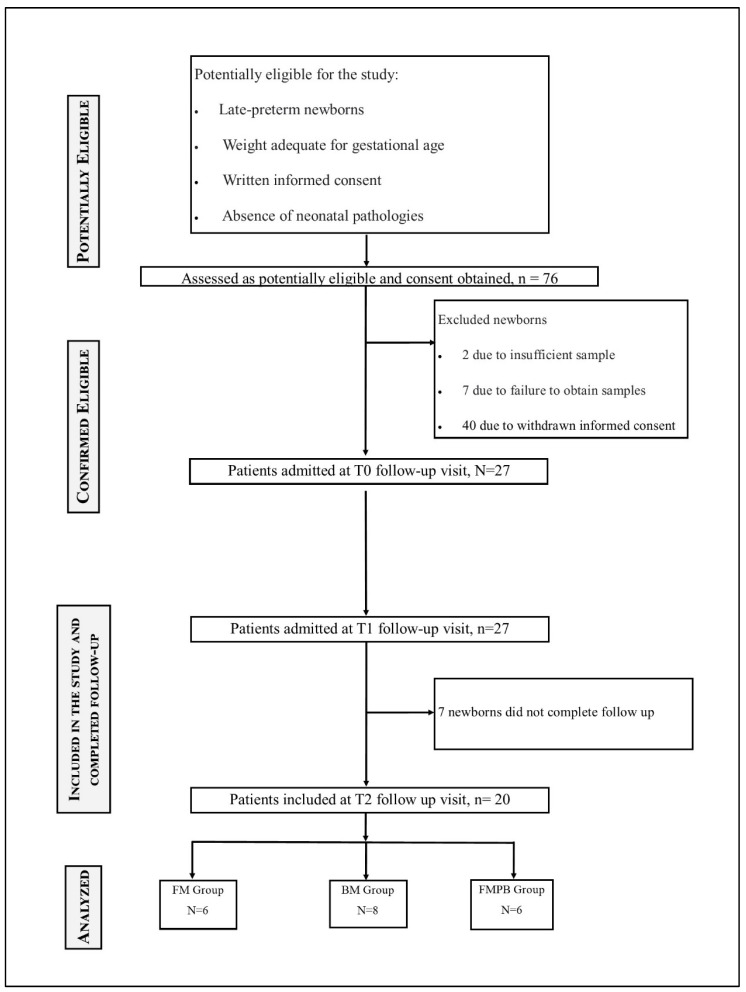
Flow-chart diagram showing the enrolment and follow-up of late preterm infants.

In the present study, a total of 76 newborns were assessed as potentially eligible. However, 27 newborns were confirmed eligible and completed follow-up at T0 and T1. At T2, 20 newborns completed follow-up analysis, as reported in the flow diagram (Figure 1). For different reasons, such as samples in small amounts, failure to collect the samples, or the informed consents, a small sample size was obtained. Many parents were unwilling to take on the responsibility of collecting, storing in the fridge, or transporting the sample to the hospital, in particular at time 2, when the subjects of this study decreased. 

### 3.1. Descriptive Data and Outcome Data

The baseline characteristics of the newborns of the gestational age, gender, delivery mode, body weight, length, head circumference, and Apgar score were not statistically different (Table 1). 

### 3.2. Metabolic Profile of Preterm Infants According to Diet: Untargeted and Targeted LC-MS Analysis

In this innovative and preliminary study, we analyzed the metabolic profile in late preterm infants, comparing two dietary regimes versus breastfeeding preterm infants.

**Figure 2:**
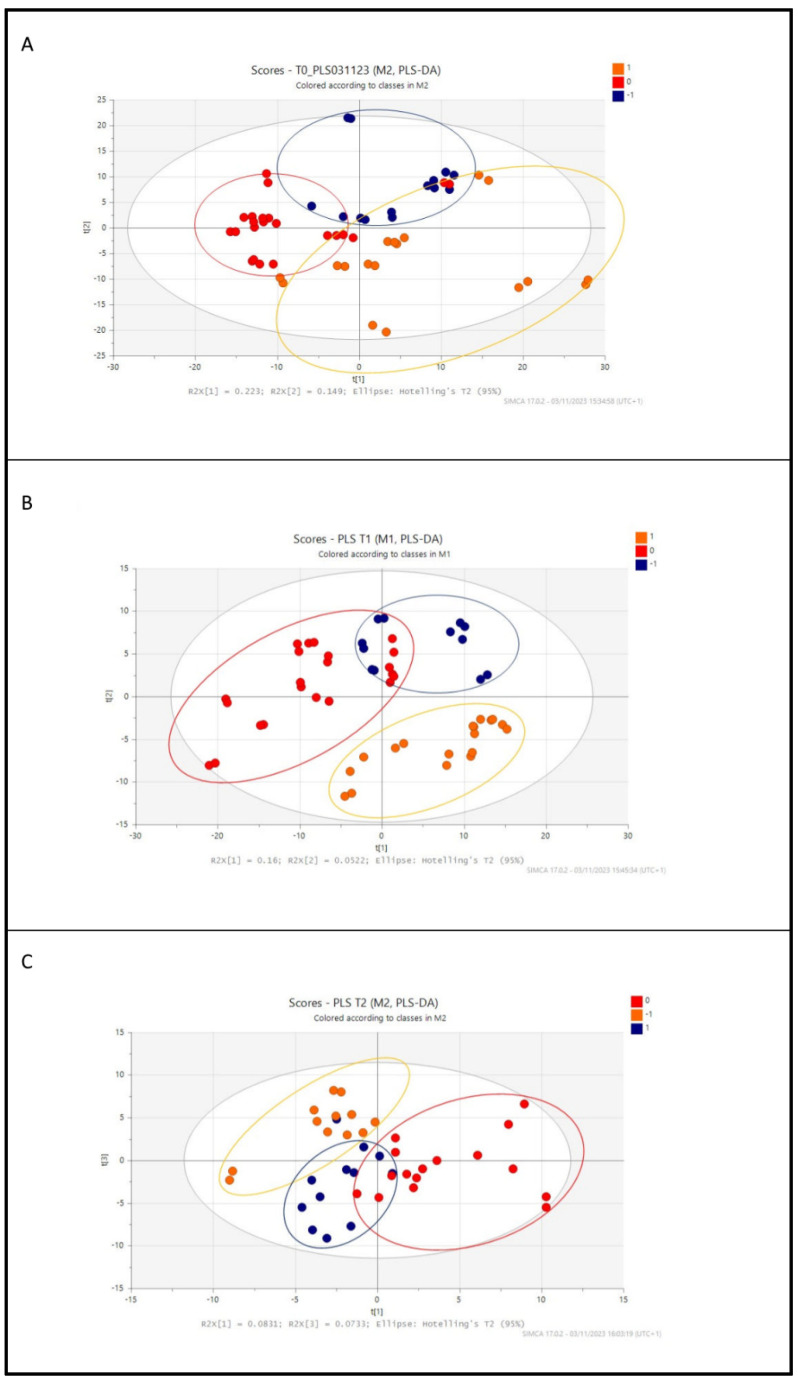
Untargeted analysis PLS-DA score scatter plot of (**A**) T0 samples, (**B**) T1 samples, and (**C**) T2 samples colored according to the type of feeding (breast milk (BM)-fed infants colored and encircled in red, formula milk (FM) colored and encircled in orange, and formula plus postbiotic (FMPB) milk colored and encircled in blue).

Stool samples were collected and submitted to solvent extraction (acetonitrile: water 2:1), centrifugation, and filtration to produce a supernatant that, after opportune dilution with water, was subjected to LC-MS and MS/MS analysis in a data-dependent acquisition mode. In the first experimental approach, an untargeted analysis was performed, and multivariate data analysis was applied to the LC-MS data acquired for the different times T0, T1, and T2 of the newborn samples (54 samples for T0 and T1, 40 samples for T2). The three generated data matrices were then processed for the Partial Least Square Discriminant Analysis (PLS-DA). The PLS-DA gave a classification model in which each sample, corresponding to breast (BM)-, standard (FM)-, or standard + postbiotic (FMPB)-fed infants, is correlated with the *m*/*z* and peak area of the metabolites found. The score scatter plots in Figure 2 clearly show a progressive similarity between the metabolic profile of the reference BM (in red)- and FMPB (in blue)-fed infants, starting from T0 to T2 (Figure 2A, Figure 2B, and Figure 2C, respectively).

We then analyzed the metabolome with a pseudo-targeted analysis approach, in a way to retrieve a list of metabolites with evident differences and/or similarities between the groups at any time of nutrition. For the pseudo-targeted analysis, exact MS and MS/MS analysis combined with a database search (Compound Discoverer software) allowed for the identification of several metabolites in all samples at different times, which were grouped under chemical classes (Tables S1–S3), mainly amino acids, bile acids, organic acids, and fatty acids derivatives. 

For the pseudo-targeted approach, new data matrices at different times (T0, T1, and T2) were obtained, considering only the known metabolites, and submitted to SIMCA software for PLS-DA analysis. 

**Figure 3:**
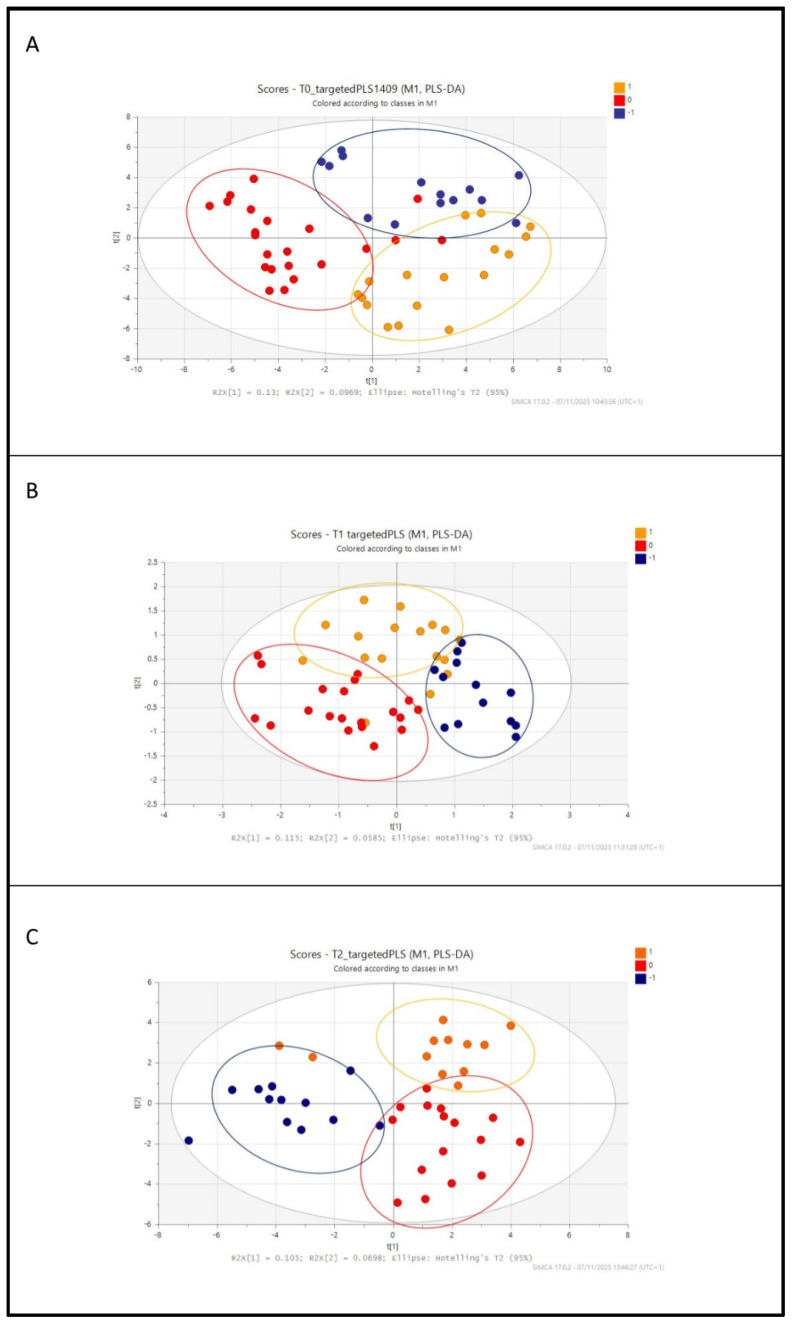
Pseudo-targeted analysis PLSDA score scatter plot of (**A**) T0 samples, (**B**) T1 samples, and (**C**) T2 samples colored according to the type of feeding (breast milk-fed infants in red, formula milk in orange, and formula plus postbiotic milk in blue).

Figure 3 shows the scatter plots for the pseudo-targeted analysis, which demonstrated a similar trend with the previous untargeted analyses. 

A clear separation between the three differently fed groups was evident already at T0, and some similarities between the reference and the FMPB groups at different times were observed. The delivery mode, such as vaginal or caesarean delivery, was not considered since most of the babies were born in urgency via caesarean delivery.

### 3.3. Analysis of the Identified Metabolites among Groups

**Figure 4:**
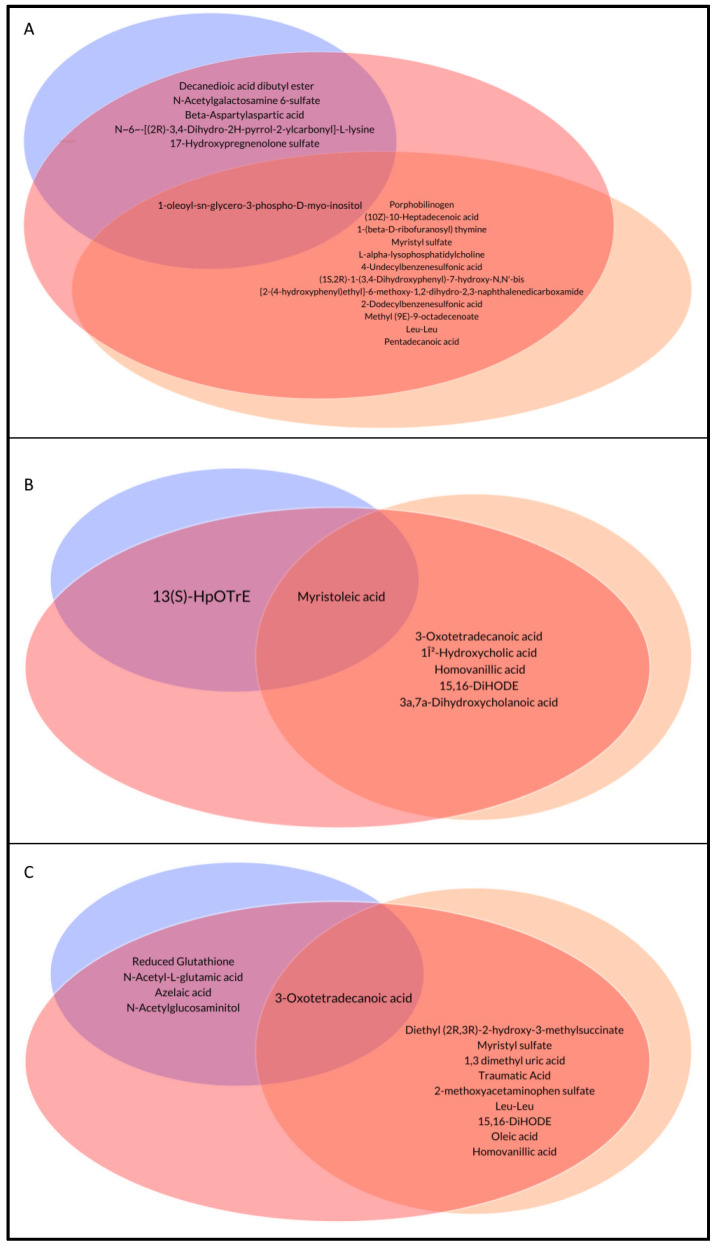
Venn diagram of common metabolites among groups at time 0 (**A**), time 1 (**B**), time 2 (**C**). Breast milk-fed infants in red, formula milk in orange, and formula plus postbiotic milk in blue.

To have a deeper insight into the metabolome composition of the three groups, the loading plots representing the graphic image of the metabolites spreading, correlated to the groups’ distribution in the score scatter plot in Figure 3, were evaluated at different times. Moreover, a statistical analysis was carried out to obtain a list of metabolites that were different and common among groups (Table 2, Figure 4). 

The analysis of the loading plots (Figure S1) and statistics showed that most of the identified metabolites were common among groups (Tables S1–S3), indicating that both formula preparations mimic breast milk. Moreover, some significant metabolites were also found to be common only between FMPB or FM compared to BM. The BM and FMPB samples shared some interesting similarities at T0 and T1 and were mainly characterized by the presence of unsaturated and saturated fatty acids, like palmitelaidic acid, myristoleic acid, (9Z,11E,15Z)-13S-hydroxyoctadeca-9,11,15-trienoic acid (13(S)- HpOTrE), palmitic acid and azelaic acid), and bile acids (e.g., taurohyocholic acid and cholic acid). Moreover, common metabolites between BM and FMPB were *N*-acetylglucosamine-6-sulfate (GlcNAc-6-S) at time T0, 13 (S)-HpOTrE at time T1, and reduced glutathione (GSH) at time T2. These metabolites were reported to be involved in interesting biological activities.

## 4. Discussion

When breastfeeding is not an option or choice for a mother, the provision of infant formula becomes the most appropriate alternative, with the goal of delivering nutritional and functional characteristics as similar as possible to those found in human milk. It has been reported that the intestinal microbiome of premature infants is influenced by the postnatal time, birth weight, gestational age, and nutrition [12]. In fact, breastfeeding supports intestinal health in preterm infants, leading to a dynamic interplay between host and dietary factors that facilitate the colonization and enrichment of specific microbiota. However, when breast milk is unavailable or insufficient to meet the infant’s nutritional requirements, formula milk is provided and utilized as an alternative. Recently, many studies conducted on infants have demonstrated that the addition of probiotics or postbiotics to formula milk is an effective alternative to breast milk with many benefits for the babies [13,14]. In the present work, a prospective observational cohort study was conducted on the stool samples of late preterm infants fed with three different diets and at T0, T1, and T2 collection times (T0: 5–7 days after birth; T1: 30 days of life; and T2: 90 days of life). This observational study enrolled a small sample size so it can be considered as a model for subsequent and more comprehensive research. Two different metabolomic approaches were sequentially applied: an untargeted analysis, to have a first metabolic distribution among the analyzed samples, and a pseudo-targeted analysis, to gain information about the chemical classes of common and specific metabolites in the three groups. In the first step, the PLS-DA score scatter plots in the untargeted analysis highlighted a good matching between the BM and FMPB groups, which increased, starting from T0 to T2. Then, a pseudo-targeted approach was carried out to find differences and similarities between the metabolic profiles of the three groups at each time. A high-resolution mass spectrometry analysis in the data-dependent mode of the collected stool samples was performed, allowing for the identification of several metabolites, mainly belonging to amino acids, bile acids, fatty acids, and other organic acid derivatives. A combined analysis of the loading plots and statistical analysis showed a good superimposition between the fecal metabolome of preterm infants fed with FM, FMPB, and BM (reference group). These results are further evidence that formula milk substitutes can be considered as a good alternative to breast milk. Moreover, the BM and FMPB samples shared some interesting similarities at T0 and T1, mainly regarding the occurrence of unsaturated and saturated fatty acids, such as palmitelaidic acid, myristoleic acid, 13 (S)- HpOTrE, palmitic acid, pentadecanoic, and azelaic acid, as well as bile acids, such as taurohyocholic acid and cholic acid. Among them, azelaic acid, also found at T2, was reported as an important marker in the modulation of colitis, and a low level was observed in the stool samples of IBD (inflammatory bowel disease) patients and in mice models [15]. In the present study, azelaic acid was found in higher amounts in the FMPB and BM compared to FM groups, suggesting a potentially beneficial effect of breast or FMPB feeding. Furthermore, *N*-acetylglucosamine-6-sulfate (GlcNAc-6-S) was found among the significant metabolites at T0, and was shown to support the growth of *Bifidobacterium breve* UCC2003, exerting a beneficial action on the intestinal walls [16]. 13(S)-HpOTrE was also found in common between BM and FMPB and reported to exert an anti-inflammatory activity by inactivating the NLRP3 inflammasome complex through the PPAR-γ pathway [17]. Interestingly, at time T2, reduced glutathione (GSH) was found in lower amounts in BM and FMPB, compared to the formula-fed infants. This metabolite is a biomarker of the antioxidant state and is the most important intracellular agent produced in erythrocytes, exerting its action via the oxidation of GSH to oxidized glutathione (GSSG) [18]. Additionally, the oral supplementation of reduced GSH has been shown to improve cachexia and growth in pediatric patients with cystic fibrosis [19]. Thus, lower amounts of GSH in the stool samples of the BM and FMPB groups may indicate a greater use by the organism and a health benefit for newborns. On this basis, the addition of a postbiotic, particularly the SMART D3 MATRIX, to a formula milk seems to be well tolerated by the infants and could be considered a good alternative to breast milk in cases where breast milk is unavailable or insufficient. These findings could represent a starting point for subsequent deeper analyses, giving a more global understanding of the effects of substitutive nutrition to breastfeeding in preterm infants.

## 5. Conclusions

For the first time, an observational cohort study was carried out on late preterm infants subjected to three distinct dietary regimens. The research underscores similarity in the fecal metabolome across FM, FMPB, and BM, the latter used as reference, indicating that formula milk partially imitates the properties of breast milk. With multivariate data analysis was possible to differentiate the three sample types, revealing a noteworthy correlation between BM and FMPB. Detailed identification of specific metabolites that predominantly characterize both BM and FMPB was achieved, with some instances involving metabolites associated with potentially advantageous biological effects. Grounded in these findings, the enhanced formula milk incorporating SMART D3 MATRIX could be deemed a practical substitute for breast milk in situations where breast milk is unavailable or insufficient. In conclusion, this preliminary investigation opens avenues for exploration and the identification of crucial insights for future research.

## Figures and Tables

**Table 1:** Baseline characteristics of the newborns.

	FM	FMPB	BM
Gestational Age	35 (34.5–35.4)	35.6 (34.3–36.6)	35.6 (34.6–36.1)
Gender (%)	F (55.6) M (44.4)	F (57.1) M (42.9)	F (63.6) M (36.4)
Delivery mode (%)	VD (11.1) CS (88.9)	VD (28.6) CS (71.4)	VD (36.4) CS (63.6)
Birth body weight (g)	2444 ± 318	2417 ± 492	2367 ± 389
Length (cm)	47 ± 2.5	45 ± 2.3	46 ± 2.26
Head circumference (cm)	33 ± 1.22	32 ± 1.49	32 ± 1.27
Apgar score 1 min	8 (7–8)	8 (8–8)	8 (8–9)
Apgar score 5 min	9 (8–9)	9 (9–9)	9 (9–10)

Data are reported as mean + standard deviation or median (interquartile range) as appropriate. All data are not significant. VD: vaginal delivery; CS: caesarean section; F: female; M: male; FM: formula milk; FMPB: formula milk + postbiotic; BM: breast milk.

**Table 2:** Significant metabolites identified in the 3 groups (FBM, BM, and FM) of late preterm infants analyzed at T0, T1, and T2.

	FMPB	BM	FM	FM vs. BM	FMPB vs. BM
T0				*p*_Value	*p*_Value
Porphobilinogen	3.95 (0.5–5.13)	0.3 (0.3–0.3)	1,55 (0.3–3.98)	0.061	0.001 *
(10Z)-10-Heptadecenoic acid	5.9 (5.5–6.13)	0.3 (0.3–5.3)	2.65 (0.3–5.5)	1	0.001 *
Decanedioic acid dibutyl ester	5 (2.7–5)	4.8 (3.25–5.5)	2.2 (0.3–4.1)	0.001 *	1
1-(beta-D-ribofuranosyl) thymine	5.55 (2.4–5.95)	0.3 (0.3–0.3)	0.3 (0.3–5.88)	0.206	0.001 *
Myristyl sulfate	4.3 (3.8–5.8)	0.3 (0.3–3.8)	2.2 (0.3–4.3)	0.09	0.002 *
L-alpha-lysophosphatidylcholine	3.25 (0.3–5.25)	0.3 (0.3–0.3)	0.3 (0.3–3.4)	0.272	0.002 *
N-Acetylgalactosamine 6-sulfate	3.95 (0.3–5.25)	5.5 (0.3–6.15)	0.3 (0.3–0.3)	0.003 *	0.414
Beta-Aspartylaspartic acid	0.3 (0.3–1.25)	0.3 (0.3–0.3)	5.4 (0.3–5.85)	0.005 *	1
4-Undecylbenzenesulfonic acid	0.3 (0.3–0.3)	0.3 (0.3–3.75)	0.3 (0.3–0.3)	0.156	0.007 *
(1S,2R)-1-(3,4-Dihydroxyphenyl)-7-hydroxy-N,N’-bis [2-(4-hydroxyphenyl)ethyl]-6-methoxy-1,2-dihydro-2,3-naphthalenedicarboxamide	6.2 (5.725–6.7)	5.6 (0.3–6.15)	4.9 (0.3–6.05)	1	0.026 *
2-Dodecylbenzenesulfonic acid	0.3 (0.3–0.3)	0.9 (0.3–4.5)	0.3 (0.3–4.6)	1	0.011 *
N~6~-[(2R)-3,4-Dihydro-2H-pyrrol-2-ylcarbonyl]-L-lysine	0.3 (0.3–4.6)	0.3 (0.3–0.3)	2.1 (0.3–5.9)	0.011 *	0.62
17-Hydroxypregnenolone sulfate	0.3 (0.3–1.675)	0.3 (0.3–0.3)	0.3 (0.3–5.5)	0.015 *	0.912
Methyl (9E)-9-octadecenoate	3.8 (0.3–5.8)	0.3 (0.3–0.3)	0.3 (0.3–5.5)	0.127	0.033 *
1-oleoyl-sn-glycero-3-phospho-D-myo-inositol	0.3 (0.3–0.3)	0.3 (0.3–3.45)	0.3 (0.3–4.575)	1	0.101
Leu-Leu	3.9 (2–4.7)	0.3 (0.3–3.3)	2.3 (0.5–5.05)	0.059	0.02 *
Pentadecanoic acid	0.3 (0.3–4.9)	0.3 (0.3–0.3)	0.3 (0.3–0.3)	0.413	0.043 *
				FM vs. BM	FMPB vs. BM
T1				*p*_value	*p*_value
3-Oxotetradecanoic acid	5.8 (0.3–6.2)	0.3 (0.3–0.3)	0.3 (0.3–0.3)	1	0.001 *
Taurohyocholic acid	0.3 (0.3–0.5)	5.7 (3.2–5.8)	2.7 (0.3–4.8)	0.023 *	0.001 *
Cholic acid	0.3 (0.3–0.3)	0.3 (0.3–5.8)	0.3 (0.3–0.3)	0.017 *	0.007 *
1Î²-Hydroxycholic acid	0.3 (0.3–0.3)	2.55 (0.3–6.05)	0.3 (0.3–0.3)	0.113	0.02 *
Myristoleic acid	5.5 (0.3–6.1)	0.3 (0.3–3.2)	0.3 (0.3–0.3)	1.000	0.067
13(S)-HpOTrE	2.6 (1.7–5.2)	4.75 (3.7–5.35)	2.6 (0.3–4.1)	0.028 *	0.388
Homovanillic acid	5.6 (0.3–6)	0.3 (0.3–0.3)	0.3 (0.3–0.3)	1	0.037 *
15,16-DiHODE	3.7 (0.3–5.1)	5 (4–6.2)	4.3 (2.2–5.1)	0.313	0.039 *
3a,7a-Dihydroxycholanoic acid	0.3 (0.3–0.3)	0.3 (0.3–6.1)	0.3 (0.3–0.3)	0.296	0.043 *
				FM vs. BM	FMPB vs. BM
T2				*p*_value	*p*_value
Diethyl (2R,3R)-2-hydroxy-3-methylsuccinate	7.3 (7.1–7.5)	6.4 (5.9–6.5)	6 (5.7–6.6)	1	0.001 *
Reduced Glutathione	3.1 (0.3–6.1)	5.1 (0.3–5.7)	6.5 (6.4–6.5)	0.001 *	1
N-Acetyl-L-glutamic acid	5.4 (3.1–6.2)	4.3 (2.3–5.9)	6.8 (6.2–7)	0.001 *	1
Myristyl sulfate	4.3 (4.3–5)	5.3 (4.8–6.3)	5.8 (5.8–5.8)	1.000	0.007 *
1,3 dimethyl uric acid	0.3 (0.3–0.3)	2.8 (0.3–4.95)	0.3 (0.3–1.7)	0.055	0.002 *
Traumatic Acid	5.7 (5.1–5.85)	3.6 (1.7–4.85)	5 (4.6–5.65)	0.058	0.003 *
2-methoxyacetaminophen sulfate	2.85 (0.85–5.9)	6.1 (5.4–6.55)	5.45 (4.65–5.6)	0.212	0.01 *
Leu-Leu	4.3 (2.4–5.5)	1.85 (0.85–3.55)	2.2 (1.3–3.2)	1	0.038 *
Azelaic acid	6.7 (6.5–7.1)	7.5 (6.7–7.5)	6.7 (5.75–6.7)	0.026 *	0.17
N-Acetylglucosaminitol	5.5 (2.05–6.15)	2.45 (0.3–5.4)	5.8 (2.75–6.5)	0.043 *	1
15,16-DiHODE	4.7 (4.15–5.3)	5.5 (4.9–6)	5.4 (5.15–5.9)	0.978	0.021 *
Oleic acid	5.8 (5.5–5.8)	6.6 (5.8–6.6)	5.8 (5.8–6.6)	1	0.035 *
3-Oxotetradecanoic acid	0.3 (0.3–4.1)	3.3 (0.3–6.25)	5.8 (5.3–6)	1	0.24
Homovanillic acid	5.8 (5.4–6.2)	2.9 (0.65–5.65)	5.3 (3.65–5.7)	0.44	0.047 *

Data are reported as median (interquartile range); IQR: interquartile range; * *p* < 0.05; FMPB: formula milk + postbiotic; BM: breast milk; FM: formula milk.

## Data Availability

The data presented in this study are available on request from the corresponding author. The data are not publicly available due to ethical restrictions.

## Supplementary Materials

The following supporting information can be downloaded at: https://www.mdpi.com/article/10.3390/metabo14010072/s1, Figure S1: Loading scatter Plot at time 0 (A), time 1 (B), time 2 (C) with variables distributed in the plot according to the distribution of samples in the score scatter plot. Table S1: common metabolites (T0) among the different diet regimens (FMPB; BM; FM) grouped based on their belonging class; Table S2: common metabolites (T1) among the different diet regimens (FMPB; BM; FM) grouped based on their belonging class; Table S3: common metabolites (T2) among the different diet regimens (FMPB; BM; FM) grouped based on their belonging class.
