# Supplementary material for: Analysis of the Fecal Metabolomic Profile in Breast vs. Different Formula Milk Feeding in Late Preterm Infants
Source: Metabolites. 2024 Jan 22;14(1):72. doi: 10.3390/metabo14010072 (PMC10820811; doi:10.3390/metabo14010072)
Supplement: Supplementary file 1 [file metabolites-14-00072-s001.zip › metabolites-2809415-supplementary.pdf]

Analysis of the Fecal Metabolomic Profile in Breast vs. Different Formula Milk Feeding in Late Preterm Infants

Giuseppe De Bernardo [1,*], Gilda D'Urso [2], Simona Spadarella [1], Maurizio Giordano [3], Giuseppina Leone [1] and Agostino Casapullo [2,*]

1 Division of Pediatrics Neonatology and NICU, Ospedale Buon Consiglio Fatebenefratelli, 80123 Naples, Italy; spadarella.simona@fbfna.it (S.S.); leone.giuseppina@fbfna.it (G.L.)
2 Department of Pharmacy, University of Salerno, via Giovanni Paolo II, 132, 84084, Fisciano, Italy; gidurso@unisa.it
3 Department of Clinical Medicine and Surgery, Federico II University, 80138 Naples, Italy; mauri.giordano@studenti.unina.it
* Correspondence: debernardo.giuseppe@fbfna.it (G.D.B.); casapullo@unisa.it (A.C.)

Supplementary Figure S1: Loading scatter Plot at time 0 (A), time 1 (B), time 2 (C) with variables distributed in the plot according to the distribution of samples in the score scatter plot.

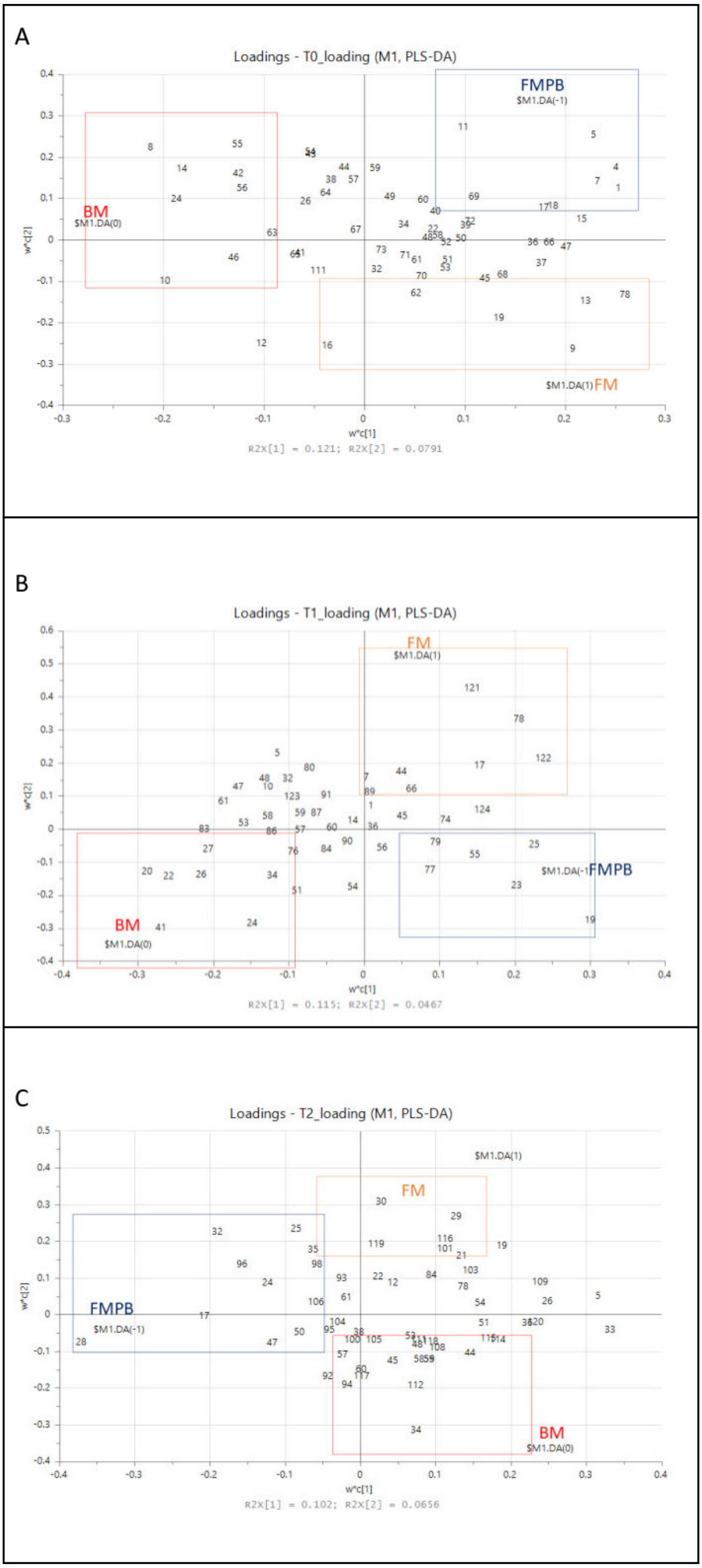

FMPB: Formula milk plus post biotic (text and rectangle blue); FM: formula milk (text and rectangle orange); BM: breast milk (text and rectangle red).

1 (Porphobilinogen); 4 (1-(beta-D-ribofuranosyl) thymine); 5 (Myristyl sulfate); 7 (L-alpha-lysophosphatidylcholine); 8 (N-Acetylgalactosamine 6-sulfate); 9 (Beta-Aspartylaspartic acid); 10 (4-Undecylbenzenesulfonic acid); 11 ((1S,2R)-1-(3,4-Dihydroxyphenyl)-7-hydroxy-N,N'-bis[2-(4-hydroxyphenyl)ethyl]-6-methoxy-1,2-dihydro-2,3-naphthalenedicarboxamide); 12 (2-Dodecylbenzenesulfonic acid); 13 (N~6~-[(2R)-3,4-Dihydro-2H-pyrrol-2-ylcarbonyl]-L-lysine); 14 (17-Hydroxypregnenolone sulfate); 15 (Methyl (9E)-9-octadecenoate); 16 (1-oleoyl-sn-glycero-3-phospho-D-myo-inositol); 17 (Leu-Leu); 18 (Pentadecanoic acid);

19 (3-Oxotetradecanoic acid); 20 (Taurohyocholic acid); 21 (Cholic acid)¸ 22 (Hydroxycholic acid); 23 (Myristoleic acid);24 (13(S)-HpOTrE); 25 (Homovanillic acid);26 (15,16-DiHODE);27 (3a,7a-Dihydroxycholanoic acid); 28 (Diethyl (2R,3R)-2-hydroxy-3-methylsuccinate); 29 (Reduced Glutathione); 30 (N-Acetyl-L-glutamic acid); 32 (Traumatic Acid); 33 (2-methoxyacetaminophen sulfate); 34 (Azelaic acid); 35 (N-Acetylglucosaminitol); 36 (Oleic acid); 37 (Oleoyl tyrosine);

38 (Phenylac-gln-OH); 39 (N-[(1S)-4-Carbamimidamido-1-carboxybutyl]asparaginyl-beta-aspartyl-L-arginine); 40 (Leu-Val); 41 (Glycocholic acid); 42 (Glycoursodeoxycholic acid 3-sulfate); 43 (Glycochenodeoxycholic acid); 44 (Citric acid); 45 (D-Gluconic acid); 46 (D-Glucopyranuronic acid); 47 (5-Methoxy-3-indoleaceate);

48 (Sebacic acid); 49 (3-oxopalmitic acid; 50 3-Hydroxysebacic acid); 51 (13S-hydroxyoctadecadienoic acid); 52 (Pinellic acid); 53 ((10E,15Z)-9,12,13-Trihydroxy-10,15-octadecadienoic acid); 54 (Palmitelaidic acid); 55 (Palmitic Acid); 56 (Traumatin); 57 (Phloionolic acid);

58 (12-HSA); 59 (9,10-Dihydroxystearic acid); 60 (Stearic acid); 61 (Myristic acid); 62 (Dimethyl (2E)-3-methoxy-4-oxo-5-[(8E,11E)-8,11,14-pentadecatrien-1-yl]-2-hexenedioate); 63 (Estriol); 64 (Androsterone sulfate); 65 (16alpha-hydroxydehydroepiandrosterone 3-sulfate); 66 (2-methoxy-17beta-estradiol 3-sulfate); 67 (Dehydroepiandrosterone sulfate);

68 (Tiliacorine); 69 (O-heptadecanoylcarnitine); 70 (Carbazeran); 71(Soyasaponin I); 72 (Tomelukast); 73 (N(4)-phosphoagmatine) ; 74 (N-Stearoyltyrosine); 76 (Diethyl tartrate); 77 (2-methylcitric acid);

78 (laurilsulfate); 79 (1,1′-monoglyceride citrate; 80 (1-stearyl estercitric acid); 83 (Juniperic acid); 84 (6-Hydroxy-5-methyl-4,11-dioxoundecanoic acid); 86 (1-linoleoyl-sn-glycero-3-phosphoethanolamine); 87 (1-Glyceryl stearate);

89 (phyllanthin) ; 90 (Pirsidomine) ; 91 (Omacetaxine mepesuccinate); 92 (L-gamma-Glutamyl-L-leucine); 93 (Fructoselysine); 94 (N-tert-Butyloxycarbonyl-deacetyl-leupeptin); 95 (3-Hydroxyhexadecadienoylcarnitine); 96 (4-(Trimethylammonio)-3-(undecanoyloxy)butanoate);

98 (7-ketodeoxycholic acid); 100 (Glucoheptonic Acid); 1011,5-Dibutyl 2-deoxy-3-C-(methoxycarbonyl)pentarate; 103 (Ascorbyl stearate); 104 (10,16-Dihydroxyhexadecanoic acid); 105 (7-Mercaptoheptanoylthreonine); 106 (1-Stearoyl-2-hydroxy-sn-glycero-3-PE);

108 (Patrinoside); 109 (Rehmaionoside C); 111 (Glucopyranosyloxyjasmonic acid); 112 ((2-Hydroxy-2-oxido-1,3,2-dioxaphospholan-4-yl)methyl palmitate); 114 (1,3-Dihydroxy-2-propanyl (9Z)-9-tetradecenoate); 115 (Istamycin B3); 116 (Bafilomycin A1); 117 (Eriojaposide B);

118 (Precorrin-5); 119 (Ubiquinol 10); 120 (D-Pantothenic acid); 121 (3-Hydroxy carbofuran); 122 (9,10-Epoxyoctadecanoic acid); 123 (1,3-Dimethyluric acid); 124 (dibutyl decanedioate)

Supplementary Table S1: Common metabolites (T0) among the different diet regimens (Formula milk plus Post biotics (FMPB); Breast Milk (BM); Formula Milk (FM)) grouped based on their belonging class.

**Metabolites**	**FMPB**	**BM**	**FM**
	**Median (25-75)**	**Median (25-75)**	**Median (25-75)**
	Amminoacids and derivatives		
**Oleoyl tyrosine**	0.3 (0.3-0.3)	0.3 (0.3-1.4)	0.3 (0.3-0.3)
**Phenylac-gln-OH**	2.95 (0.3-6.45)	0.3 (0.3-5.38)	0.3 (0.3-6.25)
**N-[(1S)-4-Carbamimidamido-1-carboxybutyl]asparaginyl-beta-aspartyl-L-arginine**	4.4 (1.9-5.8)	4.1 (2.15-5.2)	3.6 (0.3-5)
**Leu-Val**	0.3 (0.3-4.88)	0.3 (0.3-2.05)	0.3 (0.3-1.85)
	Bile acid and derivatives		
**1Î^{2}-Hydroxycholic acid**	0.3 (0.3-5.88)	2.45 (0.3-4.65)	0.3 (0.3-4.65)
**Glycocholic acid**	0.3 (0.3-1.05)	0.3 (0.3-5.25)	0.3 (0.3-5.2)
**Glycoursodeoxycholic acid 3-sulfate**	1 (0.3-4.78)	0.3 (0.3-0.3)	2.8 (0.3-6.4)
**Glycochenodeoxycholic acid**	2.75 (0.3-6.1)	0.3 (0.3-0.3)	0.3 (0.3-5.55)
	Organic acid		
**Citric acid**	5.5 (4.3-6.13)	5.3 (0.3-5.8)	5.4 (2.1-6.2)
**Traumatic Acid**	5 (2.3-5.25)	5 (3.7-5.4)	5 (3.35-5.4)
**D-Gluconic acid**	5.65 (0.3-6.03)	5.4 (5-5.45)	5.4 (0.3-6)
**D-Glucopyranuronic acid**	0.3 (0.3-5.58)	0.3 (0.3-6.5)	6.1 (0.3-6.15)
**5-Methoxy-3-indoleaceate**	2.9 (0.3-5.88)	0.3 (0.3-6.25)	0.3 (0.3-0.3)
	Fatty acid and derivatives		
**Azelaic acid**	5.65 (3.9-6.6)	5.35 (4.2-6.2)	5.8 (4.1-6.2)
**Sebacic acid**	4.3 (4.1-5.25)	4.6 (3.78-6.2)	4.1 (4-5.95)
**15,16-DiHODE**	5.2 (3.28-5.28)	4.1 (2.2-4.78)	4.7 (3.6-5.7)
**3-oxopalmitic acid**	4.8 (1.95-4.9)	2.05 (0.3-4.8)	3.3 (0.3-5.3)
**3-Hydroxysebacic acid**	5.6 (5.35-6.1)	5.6 (5.05-6.03)	5.8 (4.3-6.1)
**13S-hydroxyoctadecadienoic acid**	2.4 (2.03-5)	3.45 (1.68-5.2)	2.4 (1.4-4.55)
**Pinellic acid**	3.7 (3.65-5.2)	4.15 (2.57-5.5)	3.9 (0.3-5.2)
**(10E,15Z)-9,12,13-Trihydroxy-10,15-octadecadienoic acid**	2.1 (0.3-4.03)	2.3 (1.8-3.68)	2 (0.3-3.9)
**3-Oxotetradecanoic acid**	0.3 (0.3-4.6)	2.9 (0.3-6.2)	0.3 (0.3-2.75)
**Palmitelaidic acid**	5.1 (4.28-5.45)	3.15 (0.3-5.6)	4.8 (3.6-5.55)
**Palmitic Acid**	5 (3.9-5.4)	2.1 (0.3-5.2)	5 (3.6-5.6)
**Traumatin**	5 (0.3-5.7)	3.7 (0.3-4.35)	5.2 (3.6-5.6)
**13(S)-HpOTrE**	2.15 (0.3-5.1)	1.1 (0.3-4.5)	4.5 (2.15-5.5)
**Oleic acid**	5.8 (5.5-6.6)	6 (5.5-7)	5.8 (5.5-5.8)

**Phloionolic acid**	5 (2.55-5.83)	3.6 (1.8-5)	4.6 (2.65-5.5)
**12-HSA**	5.45 (1.93-6.4)	5.45 (2.85-6.2)	4.3 (0.4-6.2)
**9,10-Dihydroxystearic acid**	3.3 (1.28-4.05)	0.3 (0.3-2.08)	1.8 (0.3-3.6)
**Stearic acid**	5.8 (4.3-5.8)	5.1 (4.3-5.8)	4.7 (4.3-5.8)
**Myristic acid**	5.3 (4.25-6.03)	5.7 (4.9-6.35)	5.8 (5.2-6.2)
**Dimethyl (2E)-3-methoxy-4-oxo-5-[(8E,11E)-8,11,14-pentadecatrien-1-yl]-2-hexenedioate**	0.3 (0.3-5.63)	5.3 (1.65-5.65)	0.3 (0.3-6.3)
	Steroids		
**Estriol**	0.3 (0.3-1.7)	0.3 (0.3-1.7)	0.3 (0.3-6.5)
**Androsterone sulfate**	4.8 (0.3-5.8)	0.3 (0.3-4.9)	4.8 (0.3-5.7)
**16alpha-hydroxydehydroepiandrosterone 3-sulfate**	0.3 (0.3-1.63)	0.3 (0.3-3.6)	0.3 (0.3-4.8)
**2-methoxy-17beta-estradiol 3-sulfate**	4.95 (3.53-6.03)	4.8 (2.45-6.5)	3.3 (0.5-5.2)
**Dehydroepiandrosterone sulfate**	3.15 (0.3-5.2)	2 (0.3-5.2)	3.3 (0.3-5.3)
	Other organic compounds		
**Tiliacorine**	5.6 (0.3-6.2)	6 (3.3-6.23)	4.3 (0.3-5.8)
**O-heptadecanoylcarnitine**	0.3 (0.3-5.7)	0.3 (0.3-0.3)	0.3 (0.3-0.3)
**Carbazeran**	5.6 (5.1-5.8)	5.6 (5.4-5.8)	5.6 (4.8-5.9)
**Soyasaponin I**	0.3 (0.3-0.3)	0.3 (0.3-0.3)	0.3 (0.3-0.3)
**Tomelukast**	5.1 (5-5.25)	5 (3.68-5.45)	5 (2.95-5.3)
**N(4)-phosphoagmatine**	5.6 (4.6-6.4)	6.3 (4.25-6.7)	6.2 (5.8-6.6)

Data are expressed as peak rating.

Supplementary Table S2: Common metabolites (T1) among the different diet regimens (Formula milk plus Post biotics (FMPB); Breast Milk (BM); Formula Milk (FM)) grouped based on their belonging class.

Metabolites	FMPB	FM	BM
	**Median (25-75)**	**Median (25-75)**	**Median (25-75)**
	Amminoacids and derivatives		
**N-Stearoyltyrosine**	0.3 (0.3-0.3)	0.3 (0.3-0.3)	0.3 (0.3-0.3)
**Leu-Leu**	3.9 (0.3-5.2)	0.6 (0.3-4.85)	4.3 (0.9-5.1)
**Leu-Asn**	0.3 (0.3-3.9)	0.3 (0.3-5.65)	0.3 (0.3-1.6)
	Organic acid		
**Diethyl tartrate**	0.3 (0.3-0.3)	0.3 (0.3-5.2)	0.3 (0.3-0.3)
**2-methylcitric acid**	0.3 (0.3-6)	0.3 (0.3-5.5)	0.3 (0.3-0.3)
**Citric acid**	5.6 (0.3-6.4)	2.9 (0.3-6.4)	5.8 (0.3-6.1)
**D-Gluconic acid**	5.2 (0.3-6.1)	4 (0.3-6.05)	4.6 (0.3-5.8)
**laurilsulfate**	4.3 (4.3-4.7)	0.3 (0.3-4.7)	4.7 (4.3-6.2)
**porphobilinogen**	0.3 (0.3-0.3)	0.3 (0.3-0.4)	0.3 (0.3-2.7)
**Undecylbenzenesulfonic acid**	0.3 (0.3-2)	2.2 (0.3-3.75)	2 (0.3-3.6)
**1,1′-monoglyceride citrate**	0.3 (0.3-6.6)	0.3 (0.3-5.6)	0.3 (0.3-5.2)
	Fatty acids and derivatives		
**1-stearyl estercitric acid**	0.3 (0.3-0.3)	0.3 (0.3-2)	0.3 (0.3-4.3)
**Traumatic Acid**	0.3 (0.3-0.3)	0.3 (0.3-5.15)	0.3 (0.3-6)
**Azelaic acid**	0.3 (0.3-4.8)	4.35 (0.3-6.6)	0.3 (0.3-6.2)
**Sebacic acid**	0.5 (0.3-3.5)	3.05 (0.7-4.3)	2 (0.3-5.6)
**9,10-Epoxystearic acid**	5.2 (3.9-5.5)	5.35 (5.1-5.8)	3.9 (0.3-5.5)
**Dibutyl sebacate**	2 (0.3-5)	0.3 (0.3-3.15)	0.3 (0.3-2.4)
**13S-hydroxyoctadecadienoic acid**	2.2 (0.3-4.1)	3.15 (1.3-4.75)	0.7 (0.3-4.3)
**(10E,15Z)-9,12,13-Trihydroxy-10,15-octade-cadienoic acid**	0.3 (0.3-2)	4.45 (0.3-5.5)	3.5 (0.3-5)
**Palmitic Acid**	0.3 (0.3-0.3)	0.3 (0.3-0.3)	0.3 (0.3-0.3)
**Palmitelaidic acid**	4.8 (0.3-5.2)	3.7 (0.3-5.15)	0.3 (0.3-3)
**Traumatin**	5 (3.3-6)	5.2 (1.85-6)	4.8 (2.2-5.6)

**Oleic acid**	5.8 (4.3-5.8)	5.65 (4-6)	5.8 (3.9-5.8)
**Juniperic acid**	0.3 (0.3-0.3)	0.3 (0.3-5.6)	0.3 (0.3-2.7)
**Phloionolic acid**	5 (2.3-5.4)	4.75 (3.8-5.5)	5.4 (4.5-5.8)
**6-Hydroxy-5-methyl-4,11-dioxoundecanoic acid**	1.2 (0.3-4.8)	4.3 (0.3-4.8)	0.3 (0.3-4.3)
**12-HSA**	0.3 (0.3-0.3)	0.3 (0.3-6.2)	0.3 (0.3-4.7)
**9,10-Dihydroxystearic acid**	0.3 (0.3-2)	2.2 (0.3-4.1)	1.1 (0.3-4.2)
**Stearic acid**	3.6 (0.3-3.9)	4.3 (2.65-4.9)	4.2 (1.7-5.5)
**Myristic acid**	0.3 (0.3-0.3)	0.3 (0.3-5.4)	0.3 (0.3-4.8)
**Myristyl sulfate**	0.3 (0.3-0.3)	0.3 (0.3-2.3)	0.3 (0.3-4.1)
**Glycerol trihexanoate**	3.9 (0.3-5.6)	3.05 (0.3-5.5)	0.3 (0.3-4.8)
**L-alpha-lysophosphatidylcholine**	0.3 (0.3-0.3)	0.3 (0.3-0.3)	0.3 (0.3-5.4)
**1-linoleoyl-sn-glycero-3-phosphoethanolamine**	0.3 (0.3-0.3)	0.3 (0.3-0.3)	0.3 (0.3-0.3)
**1-Glyceryl stearate**	0.3 (0.3-0.3)	0.3 (0.3-0.3)	0.3 (0.3-0.3)
	Steroids		
**2-methoxy-17beta-estradiol 3-sulfate**	0.3 (0.3-0.3)	0.3 (0.3-0.3)	0.3 (0.3-0.3)
**17-Hydroxypregnenolone sulfate**	3.9 (0.3-5.4)	3.95 (0.3-5.3)	4.1 (0.3-5.6)
	Other organic compounds		
**5-Methoxy-3-indoleaceate**	0.3 (0.3-0.3)	0.3 (0.3-2.5)	0.3 (0.3-0.3)
**phyllanthin**	0.3 (0.3-0.3)	0.3 (0.3-0.3)	0.3 (0.3-6)
**Pirsidomine**	5.2 (0.3-6)	4.15 (0.3-5.8)	4.8 (0.3-5.8)
**Omacetaxine mepesuccinate**	0.3 (0.3-0.3)	0.3 (0.3-0.3)	0.3 (0.3-0.3)

Data are expressed as peak rating.

Supplementary Table S3: Common metabolites (T2) among the different diet regimens (Formula milk plus Post biotics (FMPB); Breast Milk (BM); Formula Milk (FM)) grouped based on their belonging class.

Metabolites	FMPB	FM	BM
	Median (25-75)	Median (25-75)	Median (25-75)
	Amminoacids and derivatives		
**L-gamma-Glutamyl-L-leucine**	2.9 (0.3-4.55)	2.25 (0.75-3.8)	0.3 (0.3-2.5)
**Fructoselysine**	4.5 (0.3-5.5)	1.25 (0.3-4.9)	3.4 (0.3-5.5)
**N-tert-Butyloxycarbonyl-deacetyl-leupeptin**	6 (5.65-6.2)	6.2 (5.8-6.4)	5.8 (5.8-6)
**3-Hydroxyhexadecadienoylcarnitine**	4.25 (0.3-5.35)	1.8 (0.3-4.8)	0.3 (0.3-5.15)
**4-(Trimethylammonio)-3-(undecanoyloxy)butanoate**	0.3 (0.3-4.1)	0.3 (0.3-0.3)	0.3 (0.3-0.3)
	Bile acids and derivatives		
**1Î²-Hydroxycholic acid**	1.2 (0.3-5)	0.3 (0.3-4.95)	4.05 (0.3-5)
**7-ketodeoxycholic acid**	6.2 (5.8-6.85)	6.15 (5.5-6.65)	6.45 (5.8-6.6)
**Cholic acid**	0.3 (0.3-5.35)	3.1 (0.3-5.6)	5.4 (4.4-5.65)
	Organic acids		
**Citric acid**	5.35 (3-6.2)	5.9 (4.3-6.65)	5.15 (4.3-6.2)
**Glucoheptonic Acid**	5.4 (4-6.3)	5.55 (4.75-5.8)	4.95 (2.75-6.05)
**1,5-Dibutyl 2-deoxy-3-C-(methoxycarbonyl)pentarate**	3.5 (2.75-4.9)	3.8 (2.7-4.8)	4.25 (3.7-5.2)
**D-Gluconic acid**	5.5 (5-5.8)	5.9 (5.5-6.5)	5.7 (4.85-5.8)
**5-Methoxy-3-indoleaceate**	6.2 (6.15-6.6)	6.35 (0.3-7)	6 (0.3-6.6)
	Fatty acid derivatives		
**Sebacic acid**	4.3 (4.3-4.75)	4.3 (4.3-6.2)	4.3 (4.3-4.7)
**9,10-Epoxystearic acid**	5.5 (4.7-5.5)	5.5 (5.1-5.65)	5.5 (5.1-5.8)
**Ascorbyl stearate**	3.8 (1.85-4.9)	4.8 (4.35-4.9)	4.7 (4.3-5.1)
**Dibutyl sebacate**	5 (4-5.65)	5.5 (4.7-5.8)	5.65 (4.3-6.2)
**10,16-Dihydroxyhexadecanoic acid**	5.8 (4.55-6.35)	5.35 (3.7-5.75)	5.1 (3.4-5.65)
**13S-hydroxyoctadecadienoic acid**	5.1 (4.7-5.7)	5.5 (5.3-5.7)	5.5 (4.85-5.55)
**3-Hydroxysebacic acid**	6.05 (5.3-7.4)	6.05 (4.8-6.8)	4.8 (4.8-7.1)

**7-Mercaptoheptanoylthreonine**	4.3 (0.3-5.65)	3.5 (0.3-6.25)	2.9 (0.3-4.35)
**(10E,15Z)-9,12,13-Trihydroxy-10,15-octadecadienoic acid**	4.2 (2.7-5.3)	4.7 (3.45-5.5)	4.6 (2.9-5.5)
**1-Stearoyl-2-hydroxy-sn-glycero-3-PE**	5.4 (4.95-5.8)	5.2 (0.3-5.25)	5.2 (2-5.5)
**Palmitic Acid**	5.65 (5.5-6.6)	6 (5.8-6.6)	5.85 (5.5-6.2)
**Palmitelaidic acid**	4.5 (2.95-5.35)	5.45 (4.6-5.55)	5.3 (4.8-5.7)
**13(S)-HpOTrE**	4.25 (1.95-4.65)	2.1 (1.1-2.25)	2 (1.85-4.5)
**12-HSA**	6.2 (5.25-6.4)	6.2 (5.75-7)	5.8 (4.5-7)
**9,10-Dihydroxystearic acid**	5.65 (5.5-6.6)	6 (5.8-6.6)	5.85 (5.5-6.2)
**Stearic acid**	5.6 (4.6-5.7)	5.8 (4.95-5.8)	4.95 (4.4-5.35)
**Myristic acid**	5.5 (5.2-5.9)	5.7 (5.2-5.9)	5.8 (5.5-6.05)
	Terpenes		
**Patrinoside**	4.6 (2.15-5.1)	5 (3.7-5.3)	4.9 (2-5)
**Rehmaionoside C**	0.55 (0.3-5)	5 (2.5-5.2)	5 (4.25-5.2)
	Other compounds		
**12-O-Î²-D-Glucopyranosyloxyjasmonic acid**	0.3 (0.3-5.6)	2.4 (0.3-5.2)	0.3 (0.3-5.2)
**(2-Hydroxy-2-oxido-1,3,2-dioxaphos-pholan-4-yl)methyl palmitate**	0.3 (0.3-4.3)	1.9 (0.3-4.65)	0.3 (0.3-1.9)
**2-Dodecylbenzenesulfonic acid**	3.2 (2.9-4.3)	3.7 (2.9-4.25)	4.1 (3.5-4.45)
**3-Hydroxycarbofuran**	0.3 (0.3-1)	0.3 (0.3-5.2)	4.45 (0.3-6.15)
**1,3-Dihydroxy-2-propanyl (9Z)-9-tetradecenoate**	3.6 (0.3-5.1)	5.2 (4.55-5.4)	4.35 (3-5.15)
**Istamycin B3**	2 (1.3-3.75)	4 (2.2-5.2)	4.35 (1.5-5.2)
**Bafilomycin A1**	2.4 (1.9-5)	4 (1.7-5.5)	5.2 (3.6-6.2)
**Phenylac-gln-OH**	5.7 (0.3-6.1)	4.95 (0.3-5.95)	2.9 (0.3-6.1)
**Eriojaposide B**	4.2 (3-5.65)	4.5 (3.8-5.8)	3.85 (3.1-4.4)
**Precorrin-5**	0.3 (0.3-3.9)	2.1 (0.3-5.5)	0.3 (0.3-4.8)
**Ubiquinol 10**	2.9 (0.3-5.85)	0.3 (0.3-5.8)	5.35 (2.75-5.75)
**D-Pantothenic acid**	1.5 (0.3-5.8)	5.5 (5.4-5.6)	5.5 (2.85-5.8)

Data are expressed as peak rating.
